# Adverse drug reactions associated with doxorubicin and epirubicin: A descriptive analysis from VigiBase

**DOI:** 10.1177/10781552221113578

**Published:** 2022-07-13

**Authors:** Deborah A. Matesun, Kofi Boamah Mensah, Peter Yamoah, Varsha Bangalee, Neelaveni Padayachee

**Affiliations:** ^1^ Department of Pharmacy and Pharmacology, Faculty of Health Sciences, 37707 University of the Witwatersrand, Johannesburg, South Africa; ^2^ Department of Pharmacy Practice, Faculty of Pharmacy and Pharmaceutical Science, College of Health Science, 98763 Kwame Nkrumah University of Science and Technology, Kumasi, Ghana; ^3^ Discipline of Pharmaceutical Sciences, 72753 College of Health Sciences, University of KwaZulu-Natal, Durban, South Africa; ^4^ School of Pharmacy, 549574 University of Health and Allied Sciences, Ho, Ghana; ^5^ College of Health Sciences, University of KwaZulu-Natal, Durban, South Africa

**Keywords:** Chemotherapy, epirubicin, doxorubicin, adverse drug reactions, VigiBase

## Abstract

**Background:**

Cancer is one of the leading causes of death globally. Owing to high toxicity, patients using chemotherapy drugs have a higher risk for developing adverse drug reactions (ADRs). Pharmacovigilance studies are essential in oncology to evaluate ADRs caused by anticancer drugs and improve patient safety. This study aimed to analyze serious ADRs associated with the use of doxorubicin and epirubicin reported to VigiBase.

**Method:**

All anonymized data on suspected ADRs for doxorubicin and epirubicin as ‘serious’ and ‘suspected’ or ‘interacting’ drugs between 1968 and 30 August 2021, were extracted from VigiBase. Descriptive statistics were conducted in Microsoft Excel, and data were summarized using frequencies and percentages.

**Results:**

A total of 35,620 serious individual case safety reports was analyzed. The majority of reports were from females (Dox = 61.41%; Epi = 86.56%), while the predominant age group was 45–64 years (Dox = 42.06%; Epi = 57.39%). Physicians were the more likely group to report serious ADRs (Dox = 50.03%; Epi = 34.11%). In general, Europe reported the highest for doxorubicin (38.08%), while Asia recorded the highest reports for epirubicin (53.28%). Oceania reported the least for both drugs (Dox = 0.45%; Epi = 0.04%), followed by Africa (Dox = 0.72%; Epi = 0.29%). Blood and lymphatic system disorders were the most reported serious category (Dox = 11053 [44.47%]; Epi = 6659 [61.84%]). The most common manifestations were febrile neutropenia (Dox = 10.52%) and bone marrow failure (Epi = 23.89%).

**Conclusion:**

This study provides relevant global insights into serious ADRs for doxorubicin and epirubicin. This knowledge may assist in minimizing and proactively managing ADRs. It can also inform policies to improve patients’ quality of life.

## Introduction

Cancer is a major global health burden and the second leading cause of death globally.^1^ According to the World Health Organization (WHO),^1^ about 19.3 million new cancer cases and 10 million deaths were estimated in 2020. Approximately, 70% of these deaths are found to occur in low- and middle-income countries.^1^

The increasing incidence and mortality have led to rapid developments in cancer therapies.^2^ Of these, chemotherapy has been widely explored and hence mostly used.^3^ However, its use is associated with a high incidence of adverse drug reactions (ADRs) owing to the intrinsic toxicity and narrow therapeutic index of the drugs.^4^ A study conducted by Pearce et al.^5^ showed that patients with cancer are vulnerable to ADRs, with 86% reporting at least one ADR during chemotherapy. Globally, chemotherapy-related ADRs overburden healthcare systems, contributing to mortality, hospitalizations, increased therapy costs, and reduced quality of life.^6,7^

Anthracycline drugs are a backbone in cancer chemotherapy worldwide. They have proven efficacy as single agents or in combination chemotherapy in treating several types of solid tumours and haematological malignancies, with very few unresponsive cancers.^8^ Daunorubicin and idarubicin are used for the treatment of leukaemia and lymphoma,^9,10^ while doxorubicin and its analogue, epirubicin, are not only effective against leukaemia and lymphoma but also against solid tumours of different etiologies (breast, lung, gastric, ovarian and brain cancers).^11–14^ Hence, doxorubicin and epirubicin are an integral part of many chemotherapy regimens and are the most widely used anthracyclines in clinical practice.^13,15^ The World Health Organization model essential list includes doxorubicin,^16^ while epirubicin has been adopted on the essential medicines list in countries in various regions of the world (Asia, Europe and Africa).^17–19^ Both drugs act by inhibiting deoxyribonucleic acid (DNA) and ribonucleic acid (RNA) synthesis, thereby interfering with cancer cell replication and proliferation. This consequently affects host non-cancer cells leading to ADRs ranging from mild to fatal, if not managed promptly. The commonly reported ADRs are nausea, vomiting, mucositis, alopecia and myelosuppression.^20^ Their use is also associated with severe but rare cardiotoxic risk, which often limits their long-term use.^21^ A retrospective study reported cardiotoxicity following doxorubicin use as the most serious and potentially lethal ADR, with a 26% incidence, and a one-year mortality as high as 50%.^22^ With the increasing use of these drugs, the risk of ADRs also increases. Hence, providing adequate drug monitoring is essential to enhance patient safety.

Pharmacovigilance (PV) is a tool for monitoring the safety of drugs after market approval. According to the WHO,^23^ pharmacovigilance is defined as ‘the science and activities related to the detection, assessment, understanding, and prevention of adverse drug effects or any other possible drug-related problems’. The popular Thalidomide tragedy characterized by the birth of babies with underdeveloped limbs in the 1960s^24^ highlighted the urgent need for an international drug monitoring system and resulted in the birth of global pharmacovigilance. During the clinical trials of a new drug, although a range of ADRs is identified, some ADRs only manifest after being used in larger populations.^25^ Post-marketing surveillance is therefore a key aspect of pharmacovigilance that provides an avenue through which rare and population-specific ADRs can be detected.

The WHO, in 1968, established the Program for International Drug Monitoring (PIDM), in collaboration with the Uppsala Monitoring Centre (UMC) located in Sweden.^26^ The UMC collates individual case safety reports (ICSRs) through national PV centres in various countries to a pool of electronic ADR database known as VigiBase. VigiBase is the world's largest ADR repository with over 20 million reports submitted from 143 full member countries and it plays a major role in signal detection and patient safety studies.^26^

Spontaneous reporting is the most common approach to reporting in most countries and it constitutes the vast majority of reports submitted to the VigiBase.^27^ It involves the voluntary reporting of ADRs by healthcare professionals or patients as they witness a reaction.^28^ This reporting system may be limited by under-reporting of ADRs due to failure to recognize an ADR or inadequate knowledge of reporting procedures among healthcare professionals, resulting in incomplete data and undiscovered ADR signals.^29^ Nonetheless, spontaneous reporting can contribute substantially to early signal detection, especially for rare or serious reactions when efficiently deployed.^30^

Recently, given the huge scale of the coronavirus disease (COVID-19) vaccination programme amidst the pandemic, there have been concerted efforts towards spontaneous reporting for prompt detection of vaccine safety issues. While this is a notable stride, ADR monitoring is not only important for COVID-19 vaccine safety but it should cut across all prevailing conditions and medicines used in their management. Unfortunately, PV is still lacking in many fields such as cancer chemotherapy. A previous study conducted by Baldo and colleagues highlighted under-reporting of chemotherapy-related ADRs to be a common phenomenon, especially in developing countries.^31^

In the field of oncology, where regimens are highly toxic and ADRs are often considered ‘normal’ or often confused for underlying clinical symptoms, the role of PV cannot be overemphasized.^32^ Investigating the safety profile of commonly used chemotherapy drugs in a global context would provide clinicians and policymakers with adequate knowledge needed to improve cancer patient safety.

### Study aim and objectives

This study aimed to analyze serious suspected ADRs relating to the use of doxorubicin and epirubicin submitted globally to VigiBase.

The specific objectives were:

- To describe serious ADRs for doxorubicin and epirubicin according to demographic factors (gender, age group and continent).
- To identify and quantify the top 30 reported serious ADRs for doxorubicin and epirubicin.

## Methods

### Study design

A quantitative secondary analytical method was used to conduct this study. Data on ICSRs for doxorubicin and epirubicin in VigiBase were collated, analyzed and interpreted.

### Data source

The data source utilized in this study was VigiBase, the WHO global database of ICSRs.^25^ VigiBase contains ICSR data on conventional medicines, traditional medicines (herbals), biological products and vaccines.

### Data extraction

In the published literature, we identified the two anthracyclines with a broad-spectrum anti-cancer activity as well as the most widely used in clinical practice: doxorubicin and epirubicin.^11,13–15^ The data set extracted from VigiBase contained all ICSRs for doxorubicin and epirubicin, registered by the reporter as ‘serious’ and ‘suspected/interacting’ drugs between 1968 and 30 August 2020. Each ICSR recorded in VigiBase is an anonymized report for a single individual who experienced one or more adverse reactions that may be linked to the use of one or more drugs. All ADRs in VigiBase have automatically been coded with the Medical Dictionary for Regulatory Activities (MedDRA) terminology into a System Organ Class (SOC); which provides a broad definition of the system affected, and a Preferred Term (PT); which provides a precise identification of the reaction. All drugs recorded are coded according to WHODrug terminology.^33^ The VigiBase data were electronically captured from the UMC into a Microsoft Excel spreadsheet, and each ICSR was extracted based on the following information:

- Administrative information (report date, type of report, qualification of reporter or notifier).
- Patient data (unique report identification number, reporting continent, gender, age group).
- The drug involved (name, dose, indication for use, route of administration, drug status as suspect/interacting or concomitant drug, drug start and stop dates, dechallenge and rechallenge information, information on other concomitant drugs used).
- Characteristics of the reported ADR (MedDRA System Organ Class, Preferred Term, and Low-Level Term, time of onset of reaction, outcome of reaction, and seriousness criteria of ADR).

According to the WHO an ADR is characterized as ‘serious’ if it resulted in death, is life-threatening, triggers hospitalization (or prolongation of existing hospitalization), causes a birth defect or congenital anomaly, leads to persistent incapacity or disability, or is judged clinically relevant by the physician who reports the case.^23^

### Inclusion and exclusion criteria

All ICSRs for doxorubicin and epirubicin registered as ‘serious’ and ‘suspected/interacting’ were included in this study. ICSRs with combination products (drugs containing suspect drugs in combination with other drugs), as well as case reports with demographic factors (age group, gender or continent) recorded as ‘unknown' were excluded. Suspected duplicate ADRs were also excluded.

### Data analysis

**Figure 1.**
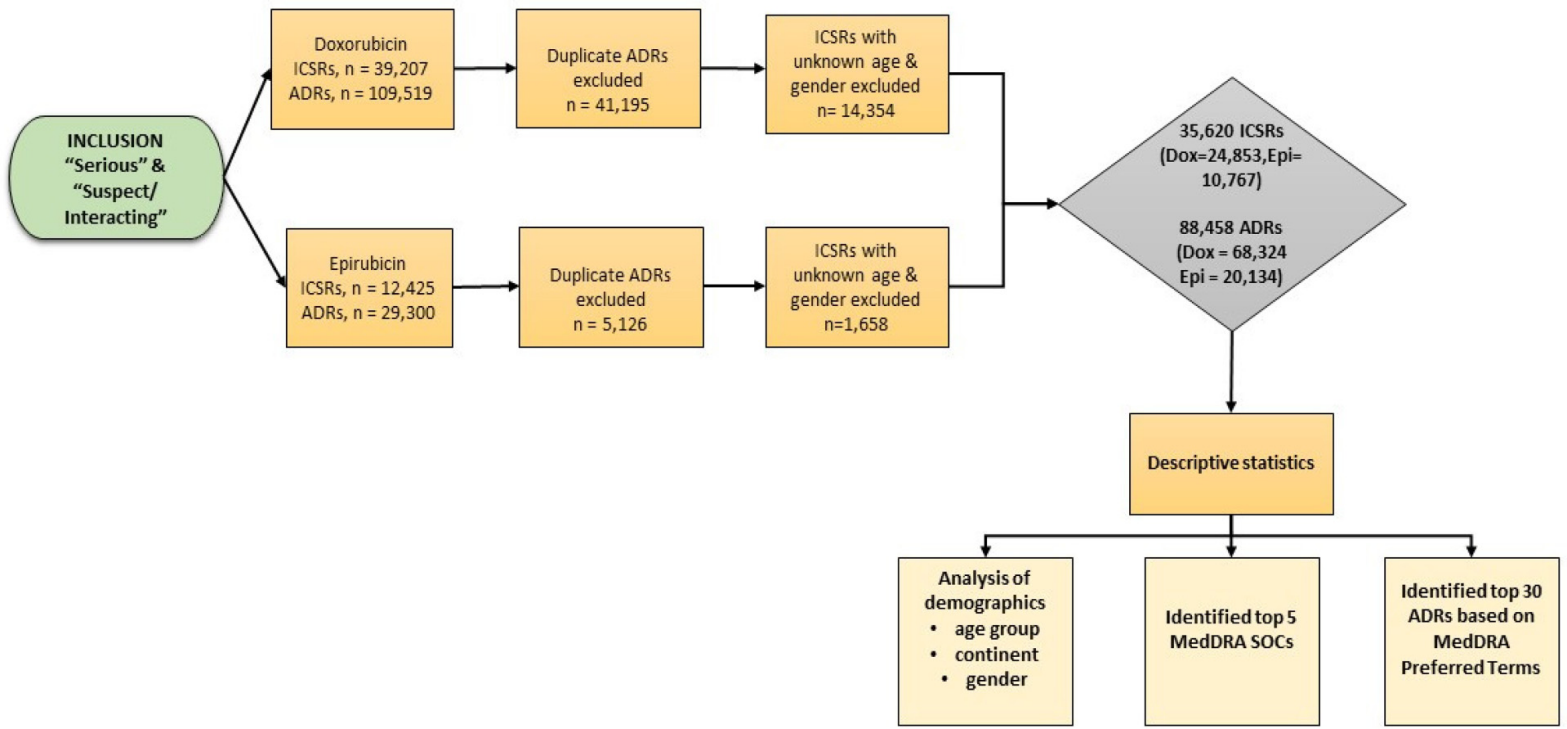
Study flow chart.

Data were cleaned and sorted, and a basic descriptive analysis was conducted using Microsoft Excel (Version 2016). ICSRs reported as ‘serious’, and ‘suspect/interacting’ for doxorubicin and epirubicin were filtered and analyzed based on demographic factors (gender, age group and continent). The top 30 serious ADRs were identified and quantified according to the MedDRA PTs using frequency tables, while the top five serious MedDRA SOCs were summarized using a frequency bar chart. The study flow chart is illustrated in Figure 1.

## Results

A total of 51,632 serious ICSRs (Dox = 39,207; Epi = 12,425) and 138,819 ADRs (Dox = 109,519; Epi = 29,300) were retrieved. Manual removal of 46,321 duplicates (i.e. reports of the same ADR sent from multiple sources) resulted in a total of 92,498 ADRs (Dox = 68,324; Epi = 24,174). After excluding ICSRs with ‘unknown’ age band and gender, 35,620 ICSRs (Dox = 24,853; Epi = 10,767) and 88,458 ADRs (Dox = 68,324; Epi = 20,134) were eligible for data analysis (Figure 1). The ADRs reported were found to be more than the ICSRs because more than one ADR can be reported for an individual patient.

### Demographic data

A total of 35,620 serious ICSRs were analyzed for both drugs. The majority of reports were from females (Dox = 61.41%; Epi = 86.56%), with the highest reports from Europe (Dox = 39.75%) and Asia (Epi = 62.96%). The predominant age group was 45–64 years (Dox = 42.06%; Epi = 57.39%), with the majority of reports from America (Dox = 37.28%) and Asia (Epi = 56.24%). Physicians were the more likely group to report serious ADRs (Dox = 50.03%; Epi = 34.11%), with Europe recording the highest number of reports (Dox = 48.87%; Epi = 82.79%). In general, Europe reported the highest for doxorubicin (38.08%) while Asia recorded the highest reports for epirubicin (53.28%). Oceania reported the least for both drugs (Dox = 0.45%; Epi = 0.04%), followed by Africa (Dox = 0.72%; Epi = 0.29%). Table 1 summarizes the demographic characteristics of serious ICSRs for doxorubicin and epirubicin.

**Table 1.** Demographic characteristics of serious individual case safety reports associated with doxorubicin and epirubicin.

Demographic characteristics	Africa		Americas		Asia		Europe		Oceania		Total	
	Dox	Epi	Dox	Epi	Dox	Epi	Dox	Epi	Dox	Epi	Dox	Epi
Gender, *n* (%)												
Female	116 (0.75)	24 (0.26)	5461 (35.78)	453 (4.86)	3552 (23.27)	4826 (51.78)	6068 (39.75)	4014 (43.07)	67 (0.43)	3 (0.03)	15264 (61.41)	9320 (86.56)
Male	63 (0.66)	7 (0.48)	3922 (40.90)	108 (7.46)	2163 (22.56)	911 (62.96)	3396 (35.41)	420 (29.02)	45 (0.47)	1 (0.07)	9589 (38.58)	1447 (13.44)
Age group, *n* (%)												
0–27 days	0 (0)	0 (0)	7 (21.21)	0 (0)	0 (0)	1 (5.56)	26 (78.79)	17 (94.44)	0 (0)	0 (0)	33 (0.13)	18 (0.17)
28 days to 23 months	1 (0.71)	1 (20)	68 (48.57)	2 (40)	28 (20)	1 (20)	41 (29.29)	1 (20)	2 (1.43)	0 (0)	140 (0.56)	5 (0.05)
2–11 years	12 (1.03)	0 (0)	551 (47.21)	4 (8.51)	244 (20.91)	23 (48.94)	357 (30.59)	20 (42.55)	3 (0.26)	0 (0)	1167 (4.70)	47 (0.44)
12–17 years	11 (1.09)	1 (2.44)	485 (47.97)	1 (2.44)	243 (24.03)	28 (68.29)	266 (26.31)	11 (26.83)	6 (0.59)	0 (0)	1011 (4.07)	41 (0.38)
18–44 years	57 (1.18)	14 (0.62)	1784 (36.79)	128 (5.64)	1242 (25.61)	1291 (56.87)	1751 (36.11)	836 (36.83)	15 (0.31)	1 (0.04)	4849 (19.51)	2270 (21.08)
45–64 years	80 (0.77)	10 (0.16)	3897 (37.28)	263 (4.26)	2568 (24.56)	3475 (56.24)	3862 (36.94)	2428 (39.29)	47 (0.45)	3 (0.05)	10454 (42.06)	6179 (57.39)
65–74 years	16 (0.31)	4 (0.22)	1831 (35.84)	121 (6.74)	958 (18.75)	721 (40.17)	2270 (44.44)	949 (52.87)	33 (0.65)	0 (0)	5108 (20.55)	1795 (16.67)
≥75 years	2 (0.10)	1 (0.02)	760 (36.35)	42 (10.19)	433 (20.70)	197 (47.82)	891 (42.61)	172 (41.75)	5 (0.24)	0 (0)	2091 (8.41)	412 (3.83)
Notifier, *n* (%)												
Physician	63 (0.50)	9 (0.24)	3847 (30.36)	169 (4.52)	2518 (19.87)	465 (12.42)	6192 (48.87)	3097 (82.79)	50 (0.39)	1 (0.03)	12670 (50.03)	3741 (34.11)
Pharmacist	74 (3.28)	13 (2.03)	512 (22.72)	28 (4.36)	596 (26.44)	145 (22.62)	1067 (47.34)	455 (70.98)	5 (0.22)	0 (0)	2254 (8.90)	641 (5.84)
Other Health Professional	38 (0.63)	8 (0.85)	3390 (56.35)	182 (19.38)	814 (13.53)	115 (12.25)	1737 (28.87)	633 (67.41)	37 (0.62)	1 (0.11)	6016 (23.76)	939 (8.56)
Lawyer	0 (0)	0 (0)	77 (71.96)	10 (18.51)	1 (0.93)	0 (0)	29 (27.10)	44 (81.48)	0 (0)	0 (0)	107 (0.42)	54 (0.49)
Consumer/Non Health Professional	6 (0.32)	0 (0)	735 (39.39)	91 (19.91)	582 (31.19)	48 (10.50)	539 (28.89)	318 (69.58)	4 (0.21)	0 (0)	1866 (7.37)	457 (4.17)
Unspecified notifier	3 (0.12)	1 (0.02)	864 (35.83)	91 (1.77)	1396 (57.90)	4982 (97.04)	131 (5.43)	58 (1.13)	17 (0.71)	2 (0.04)	2411 (9.52)	5134 (46.82)
Total reports from each continent	179 (0.72)	31 (0.29)	9383 (37.75)	561 (5.21)	5716 (23.00)	5737 (53.28)	9464 (38.08)	4434 (41.18)	111 (0.45)	4 (0.04)	24853	10767

### Frequency of serious adverse drug reactions according to system organ class involved

**Figure 2.**
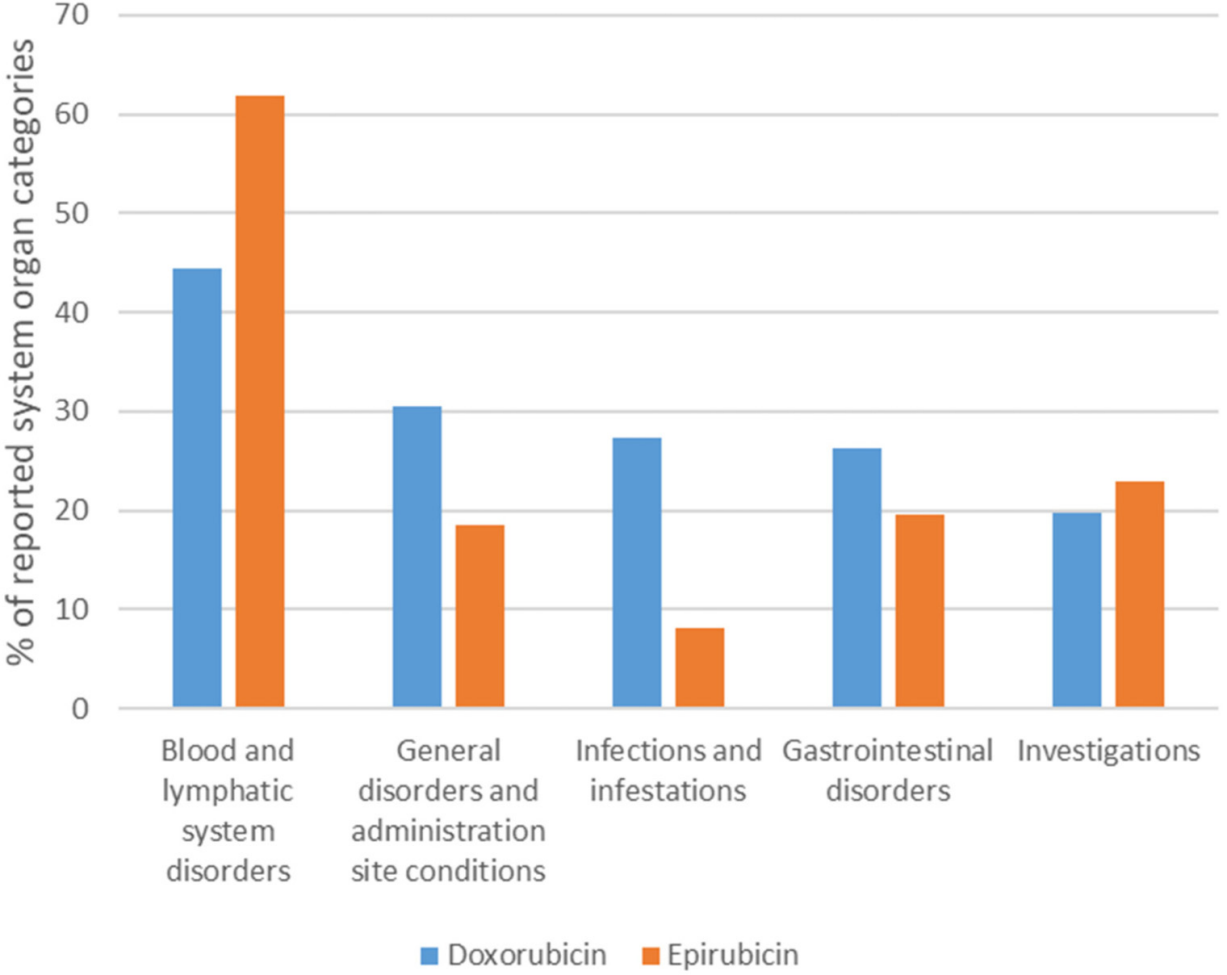
Top five serious ADRs based on system organ class for doxorubicin and epirubicin in VigiBase.

Serious ADRs identified in this study involved all the 27 MedDRA system organ classes. The top five serious system organ classes are displayed in Figure 2. The top five categories were consistent for both drugs, with blood and lymphatic system disorders being the most reported serious category (Dox = 11,053 [44.47%]; Epi = 6659 [61.84%]).

### Frequency of serious ADRs for doxorubicin and epirubicin based on MedDRA preferred terms

The frequency of the top 30 serious ADRs for both drugs is displayed in Table 2. The highest reported reaction for doxorubicin was febrile neutropenia (*n* = 2615, 10.52%) followed by neutropenia (*n* = 2370, 9.54%), while epirubicin had the highest report for bone marrow failure (*n* = 2572, 23.89%).

**Table 2.** Top 30 serious ADRs for doxorubicin and epirubicin in VigiBase.

Doxorubicin (*N* = 24,853)		Epirubicin (*N* = 10,767)	
Adverse Drug Reaction	Frequency (%)	Adverse Drug Reaction	Frequency (%)
Febrile neutropenia	2615 (10.52)	Bone marrow failure	2572 (23.89)
Neutropenia	2370 (9.54)	White blood cell count decreased	1433 (13.31)
Nausea and vomiting	2117 (8.52)	Nausea and vomiting	1118 (10.38)
Pyrexia	1532 (6.16)	Neutropenia	951 (8.83)
Anaemia	1069 (4.30)	Agranulocytosis	938 (8.71)
Off-label use/product use in unapproved indication	1040 (4.18)	Febrile neutropenia	583 (5.41)
Bone marrow failure	1013 (4.08)	Pyrexia	569 (5.28)
Leukopenia	1001 (4.03)	Leukopenia	533 (4.95)
Thrombocytopenia	982 (3.95)	Thrombocytopenia	343 (3.19)
Dyspnoea	880 (3.54)	Neutrophil count decreased	317 (2.94)
Pneumonia	835 (3.36)	Diarrhoea	293 (2.72)
Diarrhoea	784 (3.15)	Anaemia	275 (2.55)
Pancytopenia	708 (2.85)	Alopecia	221 (2.05)
Sepsis	654 (2.63)	Hepatic function abnormal	190 (1.76)
White blood cell count decreased	559 (2.25)	Dyspnoea	177 (1.64)
Fatigue	528 (2.12)	Granulocytopenia	174 (1.62)
Mucosal inflammation	526 (2.12)	Asthenia	169 (1.57)
Asthenia	519 (2.09)	Cardiac failure	163 (1.51)
Abdominal pain	484 (1.95)	Acute myeloid leukaemia	145 (1.35)
Cardiac failure	466 (1.88)	Fatigue	135 (1.25)
Alopecia	458 (1.84)	Pancytopenia	129 (1.20)
Hypotension	445 (1.79)	Pneumonia	126 (1.17)
Malignant neoplasm progression	404 (1.63)	General physical health deterioration	105 (0.98)
Palmar-plantar erythrodysesthesia syndrome	400 (1.61)	Mucosal inflammation	95 (0.88)
Neutrophil count decreased	385 (1.55)	Cardiomyopathy	91 (0.85)
Disease progression	380 (1.53)	Pulmonary embolism	84 (0.78)
Platelet count decreased	379 (1.52)	Pain	76 (071)
Acute myeloid leukaemia	374 (1.50)	Ejection fraction decreased	74 (0.69)
Cardiomyopathy	367 (1.48)	Haemoglobin decreased	72 (0.67)
Acute kidney injury	366 (1.47)	Dehydration	70 (0.65)

## Discussion

Chemotherapy is a mainstay of many clinical protocols for treating cancers globally.^3^ However, its clinical activity is limited due to the inherent toxicities of the drugs.^4^ ADRs due to cancer chemotherapy are quite challenging as they negatively impact patients’ quality of life and overburden healthcare systems.^34^ This study, to our knowledge, is the first to analyze ‘serious’ ADRs associated with two commonly prescribed anthracycline chemotherapy drugs: doxorubicin and epirubicin, from a worldwide perspective. The results from this study provide some insights into reported serious ADRs for these drugs.

Serious ICSRs contributed to over one-third (*n* = 35,620, 36%) of the total number ICSRs for both drugs. The majority of the ADRs (Dox = 61.41%, Epi = 86.56%) were reported in females, with the highest reports from Europe (Dox = 39.75%) and Asia (Epi = 62.96%). Globally, several study findings indicate that females are more prone to ADR than males.^35^ A study done in 48 communities in the United Kingdom reported a total incidence of suspected ADRs in females as 20.6 per 10,000 patient months of exposure and 12.9 per 10,000 patient months of exposure in males.^36^ Also, the results from a survey involving 10 prescription drugs withdrawn from the United States market indicated that eight of the ten drugs posed greater risks of adverse effects in women than men.^37^ Another report from the Spanish Pharmacovigilance indicates that most suspected ADRs occur in women.^38^ A review of 93 studies on ADRs associated with cardiovascular medications also reported 70% ADRs in females.^39^ Our findings may be attributed to gender-wise variations that influence drug pharmacokinetics and subsequent toxicity.^40^ For instance, due to differences in the expression levels of hepatic metabolic enzymes (CYP3A4), chemotherapy drugs tend to have a longer drug half-life and higher risk of toxicity in female patients than males.^41,42^ Other essential factors include absorption, conjugation, renal elimination, and protein binding, which may also have gender-based differences.^35^ Furthermore, studies indicate that females generally possess better healthcare-seeking attitudes compared to males, hence, they are more likely to report an ADR.^43^

The highest report of ADRs from this study was from Asia. This may be because Asia records almost half of the new cancer cases and more than 50% of the cancer mortality globally, indicating high consumption of cancer chemotherapy which may reflect more ADRs.^19^ Also, the Pan-Asia has adapted the European Society for Medical Oncology (ESMO) Clinical Practice Guidelines for the treatment of breast cancer which uses cyclophosphamide, doxorubicin and fluorouracil (CAF) and cyclophosphamide, epirubicin and fluorouracil (CEF).^18^ This indicates high consumption of anthracyclines that may reflect the high ADRs reports of the drugs recorded in Asia and Europe.

The predominant age group was 45–64 years (Dox = 42.06%, Epi = 57.39%), with the highest reports from the Americas (Dox = 37.28%) and Asia (Epi = 56.24%). Literature has shown that the prevalence of ADRs increases with age, which is consistent with our findings.^44^ This may suggest a general decline in system organ capacity leading to low metabolizing capacity and reduced excretion. Consequently, this results in the accumulation of drugs in the body, increasing the risk of ADRs.^45^ Hence, additional precautions should be taken while using chemotherapy in the elderly population. The highest ADRs recorded in the elderly were from Asia and America. Asia and North America have the highest elderly population globally, which probably may explain the results obtained.^46^

Physicians were the highest group of reporters (Dox = 50.03%, Epi = 34.11%) with the majority of the reports from Europe (Dox = 48.87%; Epi = 82.79%). A recent study in the UK revealed that consumers generally reported more ADRs; however, healthcare professionals were found to report more serious ADRs that resulted in hospitalization or caused death.^47^ Moreover, our findings may also be because only physician reporting was allowed for many years after establishing the first global ADR reporting systems in the 1960s.^48^ Only a few countries like the United States and Sweden allowed non-physician reports, and patient/other healthcare professional reporting was only incorporated over time.^48^ Till now, many countries have still not integrated consumer reporting into their national PV program.

Europe recorded the highest reports (38.08%) for doxorubicin, whereas Asia recorded the highest reports (53.28%) for epirubicin (Table 1). Oceania reported the lowest for both drugs (Dox = 0.45%, Epi = 0.04%), followed by Africa (Dox = 0.72%, Epi = 0.29%). This finding may reflect the differences in drug utilization patterns and policies across various continents of the world. For instance, the low reports for epirubicin in the Americas (5.21%) may be due to its delayed approval by the Food and Drug Administration, compared to Europe, where it gained approval since 1980.^49^ Also, previous studies have reported that epirubicin's use is favoured over doxorubicin in Asian countries, hence, the higher number of reports.^50^ The low reports from Africa are in keeping with the literature, and it depicts the state of the health systems. PV systems in Africa are weak and lack resources and infrastructure compared to other developed countries.^29^ In a global analysis conducted by Aagaard et al.,^51^ high-income countries generally reported high PV than low-income countries. Although anti-neoplastic drugs constituted the majority of the reports from high-income countries, low-income countries such as Africa reported more ADRs for anti-infectives.^51^ In line with past studies, our findings reflect a PV system in Africa that is not robust and only focuses on prevailing conditions. Therefore, efforts should be made to provide resources and infrastructure to improve PV, particularly in the African setting.

Blood and lymphatic system disorders were the predominant system organ reported (Dox = 11,053 [44.47%]; Epi = 6659 [61.84%]) showing similar ADR manifestations for both drugs. Febrile neutropenia (Dox = 10.52% of ICSRs) and bone marrow failure (Epi = 23.89% of ICSRs) were the most predominant reactions. This is in keeping with past literature as the risk of myelosuppression was found to be consistently higher in patients using anthracycline-based regimens.^52^ While destroying cancer cells, anthracyclines, are notorious for impairing rapidly dividing cells of bone marrow leading to a reduction of red blood cells, white blood cells and platelets.^53,54^ These manifestations are an important concern in clinical practice as they increase hospitalizations and the risk of infections owing to decreased immunity.^52^ Therefore, this highlights the role of healthcare professionals in identifying patients at greatest risk and minimizing the risks since long-term therapy is essential for optimal treatment outcomes.

Nausea and vomiting constituted 8.52% and 10.38% of ICSRs for doxorubicin and epirubicin, respectively. Although typically considered non-lethal, previous studies have noted these symptoms to significantly impair quality of life, and result in non-adherence or even delayed treatment due to fear.^55,56^ This is crucial as non-adherence and early discontinuation of chemotherapy have been identified as significant predictors of disease progression, and mortality in patients with cancer.^57^ Hence, early interventions may be critical to prevent and proactively manage these reactions in patients.

The observed cardiotoxicities: cardiomyopathy (Dox = 1.88%, Epi = 0.85%), cardiac failure (Dox = 1.48%, Epi = 1.51%) and decreased ejection fraction (Epi = 0.69%) are in keeping with previous studies. This may be linked to the mechanism of oxidative stress and induction of cardiac muscle cell death.^58^ McGowan et al.^21^ reported the incidence of myocardial damage from doxorubicin ranges from 1% to 20%. According to the literature, epirubicin has a lower cardiotoxic risk than doxorubicin.^12^ However, our findings could not particularly suggest if epirubicin is safer than doxorubicin since reports could have been influenced by variations in drug use and reporting biases. Baseline cardiac monitoring for patients undergoing treatment with both drugs is critical to ensure the benefits of drug use outweigh the risks. Likewise, children and adolescents should receive periodic cardiac evaluations since they have a high risk of delayed cardiotoxicity.

An interesting observation in this study is the ‘off-label use’ of doxorubicin (*n* = 1040, 4.18%). This suggests drug use in different populations, indications, or doses other than those for which it was licensed.^59^ In the literature, the off-label use of doxorubicin includes management of advanced endometrial carcinoma, metastatic hepatocellular cancer, multiple myeloma and advanced renal cell carcinoma.^14^ Although its use may sometimes be clinically justified in oncology, a major drawback is a concern about patient safety, especially for drugs that have a high potential for toxicity like chemotherapy drugs. In a study conducted in Italy, off-label use significantly increased the risk of serious ADRs in patients.^60^ Further studies may be required to re-evaluate the off-label use of doxorubicin, its harms and benefits, and regulatory actions should be taken where necessary.

Respiratory ADRs are another interesting and important set of findings from this study: pneumonia (Epi = 1.17%), pulmonary embolism (Epi = 0.78%) and dyspnoea (Dox = 3.54%, Epi = 1.64%). Pneumonia has been reported to cause or complicate almost 10% of hospital admissions among cancer patients.^61–63^ Past studies have suggested a link between epirubicin and pneumonia. For instance, Wijaya et al.^64^ observed a high incidence of *pneumocystis jirovecii* pneumonia in metastatic breast cancer patients receiving weekly epirubicin-based regimens. Also, recently during a process of evaluating serious adverse event reports, the Korean Institute of Drug Safety (KIDS) found a causal association between epirubicin and *pneumocystis jirovecii* pneumonia which led to an update of the drug label (Pharmorubicin®).^65^ Additional investigations may be required to identify patients at greatest risk who may benefit from antibiotic prophylaxis. Although there is evidence for pneumonia associated with epirubicin in the literature, there is a dearth of data regarding pulmonary embolism and dyspnoea relating to anthracycline use and these could be potentially fatal signals requiring further assessment.

### Study limitations

The main strength of this global study is the use of VigiBase, covering many countries, involving diverse clinical settings and medical cultures over an extensive period with a large sample of data. However, data reported in VigiBase might be influenced by differences in health systems, drug utilization patterns and policies over time and across various countries. Moreover, the data were retrieved according to continents, and it does not reflect which countries are making significant contributions. Hence, country-specific deductions could not be made. Also, it was not possible to measure the magnitude of risk in VigiBase due to the significant under-reporting of cases. Additionally, the likelihood that the medicine caused the reported reactions varies from report to report.^66^ Hence, further causality studies may be needed. Lastly, the data from this study lack complete clinical background information, such as existing patient co-morbidities and this may have hindered a comprehensive interpretation of the study findings.

## Study contribution

Although some of the ADRs in this study are known, these findings have highlighted the most common serious reactions reported globally on the Vigibase database. Furthermore, ADRs such as dyspnoea and pulmonary embolism have rarely been described in literature yet featured in the top 30 ADRs in the Vigibase database. Hence, this study is a call for healthcare professionals to be vigilant in monitoring and reporting ADRs, and to take proactive measures to minimize these risks in order to improve patients’ quality of life and treatment outcomes. Future PV studies may also be needed to further characterize the extent of the seriousness of the reported adverse reactions.

## Conclusion

This study provides relevant global insights into the ADRs reported for doxorubicin and epirubicin. The adverse reactions were described based on demographics and the two oncology medicines were classified in terms of their impact on organ systems, with blood and lymphatic system disorders being the most commonly reported. In addition, the top 30 reported serious ADRs were identified and quantified and the most common manifestations were febrile neutropenia and bone marrow failure. The findings from this study can assist healthcare professionals in early ADR identification and proactive risk management. This study can also inform policies in improving cancer patient safety.
